# *In vitro* IL-15-activated human naïve CD8+ T cells down-modulate the CD8β chain and become CD8αα T cells

**DOI:** 10.3389/fimmu.2024.1252439

**Published:** 2024-06-05

**Authors:** André J. Esgalhado, Débora Reste-Ferreira, Sandra Weinhold, Markus Uhrberg, Elsa M. Cardoso, Fernando A. Arosa

**Affiliations:** ^1^ Health Sciences Research Centre, University of Beira Interior (CICS-UBI), Covilhã, Portugal; ^2^ Institute for Transplantation Diagnostics and Cell Therapeutics, Medical Faculty, University Hospital Düsseldorf, Heinrich-Heine-University, Düsseldorf, Germany; ^3^ School of Health Sciences, Polytechnic of Guarda (ESS-IPG), Guarda, Portugal; ^4^ Faculty of Health Sciences, University of Beira Interior (FCS-UBI), Covilhã, Portugal

**Keywords:** IL-15, naïve CD8+ T cells, effector-memory CD8+ T cells, CD8β chain, downregulation, CD8α chain

## Abstract

Antigen-driven human effector-memory CD8+ T cells expressing low levels of the CD8β chain have been previously described. However, little is known on a possible antigen-independent trigger. We have examined the impact that IL-15 has on the expression of CD8β on purified human naïve CD8+ T cells after CFSE labeling and culture with IL-15. As expected, IL-15 induced naïve CD8+ T cells to proliferate and differentiate. Remarkably, the process was associated with a cell-cycle dependent down-modulation of CD8β from the cell surface, leading to the generation of CD8αβ^low^ and CD8αβ^−^ (i.e., CD8αα) T cells. In contrast, expression of the CD8α chain remained steady or even increased. Neither IL-2 nor IL-7 reproduced the effect of IL-15. Determination of mRNA levels for CD8α and CD8β isoforms by qPCR revealed that IL-15 promoted a significant decrease in mRNA levels of the CD8β M-4 isoform, while levels of the M-1/M-2 isoforms and of CD8α increased. Noteworthy, CD8+ T cell blasts obtained after culture of CD8+ T cells with IL-15 showed a cell-cycle dependent increase in the level of the tyrosine kinase Lck, when compared to CD8+ T cells at day 0. This study has shown for the first time that IL-15 generates CD8αα^+^αβ^low^ and CD8αα^+^αβ^−^ T cells containing high levels of Lck, suggesting that they may be endowed with unique functional features.

## Introduction

Human effector-memory CD8+ T cells (CD8+ Tem) have been thoroughly studied regarding their origin, phenotype and function. They are usually CD28−CCR7−CD45RA− but under certain conditions can re-express CD45RA, being designated as effector memory CD8+CD45RA+ T cells (CD8+ Temra) (1–3). An interesting study by Werwitzke et al., showed that about 80% of human peripheral blood CD8+ Tem cells re-express CD45RA and display low levels of the CD8αβ receptor, hence being CD28^−^CD45RA^+^CD8αβ^low^ (4). Interestingly, the phenotypic similarities between CD8+ Temra and effector-memory CD8αβ^low^ T cells also include expression of a diverse array of activation and inhibitory receptors, and secretion of IFN-γ, perforin and granzymes upon activation (4–6), suggesting that they are similar CD8+ T cell populations. Noteworthy, these studies identified a population of CD8αα^+^αβ^−^ T cells (i.e., CD8αα) in the peripheral blood of healthy and HIV-infected humans and endowed with functional effector-memory features (4–7). Konno et al. suggested that the CD8αα T cells were descendants of clonally expanded CD8αβ^high^ T cells *in vivo*, while Walker et al. suggested that the CD8αα T cells may represent circulating mucosal associated invariant T cells. By performing a TCRVβ CDR3-spectratyping analysis, the study of Konno et al. showed that CD8αβ^high^ T cells were polyclonal, while CD8αβ^low^ and CD8αα T cells were oligoclonal, suggesting that a fraction of CD8αβ^high^ T cells clonally expanded, differentiated, and downregulated the CD8β chain (5).

Besides identifying functional populations of human CD8+ T cells, the β chain of the CD8αβ receptor plays an important role in assisting CD8αβ-mediated signaling and activation mediated by the TCR. Thus, the CD8β chain is thought to increase the efficiency of the *trans*-interaction between the TCR and MHC class I molecules, allow CD8αβ heterodimers to move into lipids rafts, and enhance the activation of the fraction of Lck molecules associated with the CD8α chain (8–12). The identification that the human CD8β gene encodes four alternatively spliced membrane-bound forms (M-1, M-2, M-3 and M-4) and two secreted forms (S-1 and S-2) (13, 14) introduced more complexity into this area. Indeed, the CD8β isoforms were shown to be differentially expressed by CD8+ T cell populations, with the M-1 isoform being mainly expressed in naïve CD8+ T cells (Tn) and the M-4 isoform being predominantly expressed in CD8+ Tem (15). Moreover, mutational and functional studies with co-transduced human CD4+ T cells showed that expression of the M-4 isoform enhanced TCR-mediated recognition of antigen (16), reinforcing the importance of the CD8β chain in CD8+ T cell activation.

Despite the body of knowledge supporting the importance of the CD8β chain and the tyrosine kinase Lck in CD8+ T cell signaling and responses, studies examining the influence of factors other than peptide antigens on the expression of the CD8β chain and the tyrosine kinase Lck in human CD8+ T cells are lacking. Thus, while human CD8+ Tem cells that have reexpressed CD45RA (i.e., CD8+ Temra) have been shown to be generated by TCR-dependent and independent (i.e. cytokines) signals, such as IL-15 both *in vitro* (17–20) and *in vivo* (21, 22), human CD8αα T cells are only known to be generated by TCR-dependent signals (23). Whether IL-15 plays a role in the formation of human CD8αα T cells is not known. Prompted by our interest in characterizing molecular cues leading to the generation of CD8+ Temra cells and CD3+CD8αβ^low^ T cells, due to their association with chronic inflammatory disorders, including neurodegenerative disorders (3, 24), we used ex vivo human CD8+ T cells from the peripheral blood of regular healthy blood donors and cultured them *in vitro* in the presence of IL-15, and other members of the gamma common (γc) chain-dependent cytokines such as IL-2 and IL-7. The results demonstrated that IL-15, but not IL-2 or IL-7, induces a cell-cycle dependent downregulation of the CD8β chain, generating pools of CD8αβ^low^ and CD8αα T cells, and a marked increase in the amount of total Lck.

## Material and methods

### Ethics statement

Human peripheral blood mononuclear cells (PBMC) were obtained from buffy coats of anonymous healthy regular blood donors kindly provided by the Centro do Sangue e da Transplantação de Coimbra (CST-C, Portugal) under a protocol approved by the Portuguese Institute of Blood and Transplantation (IPST, IP, Lisbon), the University of Beira Interior (UBI), and the Faculty of Health Sciences (FCS-UBI). The study protocol (INSIGHTHEM) was approved by the Ethics Committee of the University in accordance with the Declaration of Helsinki (Ref. Number CE-UBI-Pj-2017–012). PBMC were also obtained from the Blutspendezentrale at the University Hospital Düsseldorf under protocol accepted by the institutional review board at the University of Düsseldorf (study number 2019–383).

### Isolation of cells

PBMC were isolated from buffy coats after centrifugation over Lymphoprep (STEMCELL Technologies, France). Contaminating red blood cells were lysed in lysis solution (10 mM Tris, 155 mM NH_4_Cl, pH 7.4) for 10 minutes at 37°C. Total and naïve CD8+ T cells were obtained from PBMC preparations by negative selection using MojoSort kits (BioLegend, USA). Purity was checked by flow cytometry using antibodies against CD3, CD4, CD8α, CD8β, CD45RA, CCR7, and was always higher than 96–97%.

### CFSE labeling and cell culture conditions

Freshly collected PBMC or isolated CD8+ T cells were immediately used for all studies. Cells were first labeled with CellTrace™ CFSE Cell Proliferation kit (Thermo-Fisher Scientific, USA) at a final concentration of 5μM for 5 minutes at room temperature (RT) in phosphate-buffered saline (PBS) with occasional mixing, followed by three washes with RPMI-1640 medium (Thermo-Fisher Scientific) containing 10% heat-inactivated fetal bovine serum (FBS). Then, CFSE-labeled PBMC (1.0x10^6^/mL), total CD8+ T cells (1.0x10^6^/mL) and naïve CD8+ T cells (1.5x10^6^/mL) were cultured in 24-well plates (Greiner Bio-One, Austria) in RPMI-1640 GlutaMAX medium (Thermo-Fisher Scientific) supplemented with 5% human serum (Sigma-Aldrich, USA) and 1% antibiotic-antimycotic solution (Sigma-Aldrich) at 37°C, 5% CO_2_, and 95% humidity for 12 days. PBMC and total CD8+ T cells were cultured in the presence of IL-15 (R&D Systems, USA), whereas naïve CD8+ T cells were cultured in the presence IL-15, IL-2 (Clinigen, Germany), IL-7 (R&D Systems), and combinations of IL-15+IL-2 and IL-15+IL-7. All cytokines were added at the beginning and at the sixth day of culture at a final concentration of 10ng/mL.

### Flow cytometry studies

For cell surface staining, approximately 0.5x10^6^ cells were incubated in 96-well round-bottom plates or 5mL round-bottom tubes at 4°C in the dark for 30 minutes with combinations of different fluorochrome-conjugated antibodies in staining solution (PBS, 0.2% BSA, 0.1% NaN_3_). **Supplementary Table 1** summarizes the antibodies used in this study. After staining, cells were washed, and a minimum of 20,000 events were acquired in a BD Accuri C6 (BD Biosciences, USA) or in a CytoFLEX flow cytometer (Beckman Coulter, USA). For intracellular staining, cells were first incubated with antibodies against cell surface receptors, as described. Then, cells were fixed for 30 minutes and permeabilized using the eBioscience™ Intracellular Fixation & Permeabilization Buffer Set (Thermo Fisher Scientific). After washing, cell aliquots were stained separately with irrelevant or anti-Lck antibody (see **Supplementary Table 1**) for 30 minutes at room temperature. After the washing steps, cells were resuspended in PBS and a minimum of 20,000 events were acquired in a BD Accuri C6 (BD Biosciences). For all acquisitions, doublets were excluded by FSC-A vs FSC-H. Results for extracellular and intracellular stainings were analyzed using BD Accuri C6 software (BD Biosciences), FlowJo software (FlowJo, LLC) or Kaluza Analysis Software (Beckman Coulter).

### Quantification of cell divisions

Cell divisions were determined by sequential halving of CFSE fluorescence intensity after the period of culture. In all the experiments performed, CFSE halving allowed to distinguish the different cell division cycles undergone by the CD8+ T cells. To quantitate changes in the expression of selected cell surface receptors (i.e., CD8α, CD8β, CCR7 and CD45RA) during the proliferation process electronic regions were created around positive cells in each cycle of cell division and mean fluorescence intensity (MFI) values were obtained. (see **Supplementary Figure 1**). MFI values of cells that did not divide were used to normalize the MFI values of the dividing cells as follows: (MFI dividing cells/MFI non-dividing cells) x 100. For each receptor, MFI was measured on positive cells, gated within each cell division (from 0 to 5, or more).

### RNA isolation, cDNA synthesis and qPCR

Total RNA was isolated from CD8+ T naïve cells prior and after culture with IL-15 using the RNeasy Mini Kit (Qiagen, Germany), according to the manufacturer’s instructions. All samples were treated with DNase I stock solution (Qiagen) as recommended by the manufacture. RNA quantification was performed using UV/Vis Nanophotometer spectrophotometer (Implen GmbH, Germany) and the purity was assessed using A260/280 ratio. The integrity of total RNA was based on visualization of 28S and 18S ribosomal RNA subunits under 1% agarose. Band intensity was assessed using gel documentation system 2000 (Bio-Rad, Germany). cDNA was synthesized from 0.5 or 1 μg total RNA, in a total reaction volume of 20 μl, using the Xpert cDNA Synthesis Kit (Ref. GK80.0100, GRiSP Research Solutions, Portugal) with Oligo(dT)20 primer according to the manufacturer’s instructions. cDNA was either immediately used as template in qPCR or stored at -20°C prior to qPCR. Regarding qPCR, for each assay, 100 ng of cDNA (2 μl or 1 µl, depending on if cDNA was prepared with 0.5 or 1 μg, respectively) was mixed in 20 µl final reaction mixed of a solution containing 10 µl of NZYSupreme qPCR Green Master Mix (2x), ROX plus (Ref. MB44002 NZYTech, Portugal), 0.4 µM of each primer (**Supplementary Table 2**) and sterile water. All primers were evaluated and/or designed with NCBI Primer Blast and synthesized by STAB VIDA (Portugal). Real-time PCR reactions were settled with an initial denaturation and polymerase activation at 95°C for 5 minutes, followed by 40 cycles of 95°C for 15 seconds denaturation and 60°C for 1 minute annealing/extension. All reactions were run in duplicates in 0.2 ml non-skirted 96-well PCR plates (Ref. AB0600, Thermo Fisher Scientific) with adhesive PCR plate seals (Ref. AB0558, Thermo Fisher Scientific). Negative controls without any template were processed in parallel and did not result in any qPCR signal. Specificity of primer pairs was verified by electrophoresis on a 2% (w/v) agarose gel in the presence of 0.05% Xpert Green DNA Stain (Ref. GS01.0001, GRiSP Research Solutions). The expected product size was confirmed by using the GRS Low Range Ladder Ref. GL011.0050, GRiSP Research Solutions). Standard curves were generated based on a five-fold dilution replicates series (without dilution, 1:5, 1:25 and 1:125) of a pool of different cDNAs (control and activated) analyzed in this work. Amplification efficiencies and correlation coefficients for each primer pair were calculated from the slopes of the standard curves using Excel. The relative mRNA expression of CD8α and CD8β were normalized using the measured expression level of two reference genes (GAPDH and RPS18), that were not regulated in the biological system, using the ΔCq method: relative quantity reference/target = 2^(Cq(geometric mean of reference genes)-Cq(gene of interest))^. The reference stability values for the reference genes were calculated using the CFX Maestro Software version 2.2 (Bio-Rad), according to the geNorm algorithm (25).

### Statistical analysis

For flow cytometry data, statistical analysis was performed using SPSS 28 software (IBM, USA). Continuous variables were expressed as the Mean ± Standard Error of the Mean (SEM). Differences between the means of two continuous variables were analyzed using the paired samples T-test, whereas differences among the means of three or more continuous variables were analyzed using the one-way analysis of variance (ANOVA) followed by Tukey’s or Dunnett’s *post hoc* tests. All data were checked for normality. Statistical significance was defined as p<0.05. For the qPCR data, comparison of relative quantity between control and activated cells was performed using Mann-Whitney U test, and the differences were regarded as significant when p<0.05. Statistical analysis was performed using GraphPad Prism 8.0 (GraphPad software Inc., USA).

## Results

### IL-15 induces CD8β chain down-modulation

**Figure 1:**
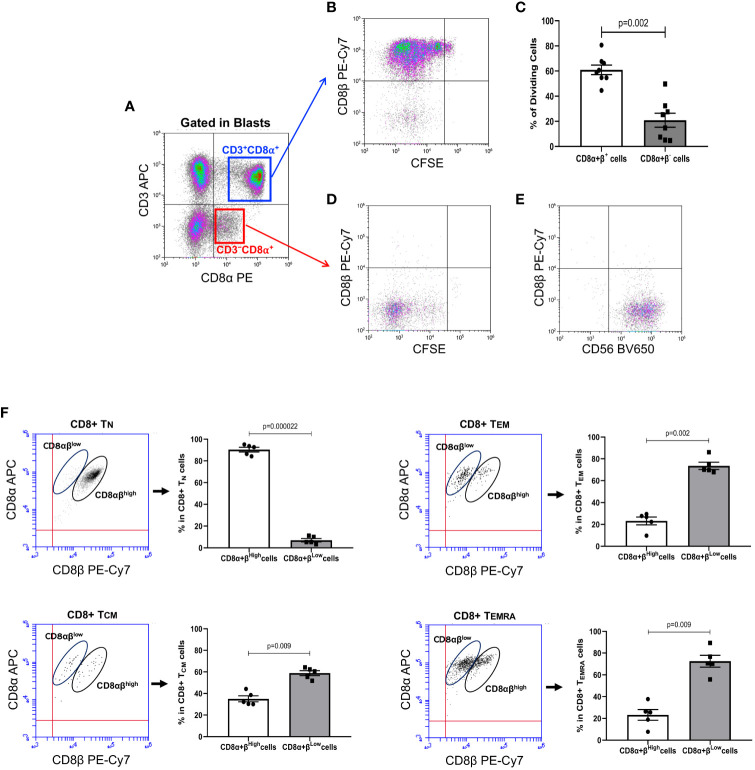
IL-15 induces down-modulation of CD8β in CD8+ T cells present in PBMC samples. PBMC were isolated from buffy coats from regular healthy donors by centrifugation over Lymphoprep, labeled with CFSE and cultured with IL-15 (10 ng/mL) for 12 days. At the end of the culture, cells were harvested, washed, and approximately 0.5×10^6^ cells stained with fluorochrome-conjugated antibodies against CD3, CD8α, and CD8β (Accuri C6) or CD3, CD8β, and CD8α, CCR7, CD45RA and CD56 (CytoFLEX) and acquired. An electronic region was created around blast cells based on FSC and SSC, which were subsequently analyzed for CFSE fluorescence halving and cell surface expression of CD3, CD8α, CD8β, CD45RA, CCR7 and CD56. **(A)** Representative dot-plot showing CD3 vs. CD8α expression on blast cells after the 12-day culture period with IL-15. Electronic gates were created around CD3+CD8α^+^ and CD3−CD8α^+^ cell blasts. **(B)** Dot-plot shows CFSE halving vs. CD8β expression after gating in CD3+CD8α^+^ T cell blasts (upper right quadrant in Panel **(A)**. This gives us an unambiguous fluorescence emission of CD8β along the cycles of cell division. **(C)** Graph showing the percentage of dividing CD8αβ^+^ and CD8αβ^−^ T cells after 12-day culture with IL-15 (mean ± SEM, n=8). **(D)** Dot-plot shows CFSE halving vs. CD8β expression after gating in CD3−CD8α^+^ T cell blasts (lower right quadrant in Panel **(A)**. **(E)** Dot-plot shows CD8β vs. CD56 expression after gating in CD3−CD8α^+^ T cell blasts (lower right quadrant in Panel **(A)**. **(F)** Figure shows dot-plots of CD8β vs. CD8α expression in CD8+ Tn, Tcm, Tem and Temra in gated CD45RA+CCR7+, CD45RA−CCR7+, CD45RA−CCR7− and CD45RA+CCR7−, respectively, after labeling of fresh PBMC preparations. CD8β^high^ and CD8β^low^ populations in each subset are circled. Next to each do-plot is a graph displaying the percentage of CD8β^high^ and CD8β^low^ T cells in CD8+ Tn, Tcm, Tem or Temra cells (mean ± SEM, n=5). P values are indicated.

In agreement with our previous results, long-term culture of human PBMC with IL-15 induced several cycles of CD8+ T cell division, as determined by CFSE halving. Noteworthy, analysis of the expression of CD8β after gating on the pool of CD3+CD8α^+^ T cell blasts (**Figure 1A**, upper right quadrant), showed an evident down-modulation of the CD8β chain after several cycles of cell division, which led to the generation of a population of CD3+CD8αβ^−^ (i.e., CD8αα) T cells in the most dividing CD8+ T cells (**Figure 1B**). Analysis of the percentage of dividing CD8αβ^−^ versus dividing CD8αβ^+^ T cells revealed that approximately 20% of the CD3+CD8αβ^+^ T cells that expanded lost expression of CD8β, becoming CD8αα T cells (**Figure 1C**). However, as expected, it was the pool of CD3−CD8+ blast cells (**Figure 1A**, lower right quadrant) that took most advantage of the presence of IL-15 in culture. Thus, as shown in **Figure 1D**, the proliferating lymphocytes were almost completely negative for CD8β expression and were largely comprised on NK cells, as demonstrated by CD56 expression (**Figure 1E**). Next, we wanted to analyze the expression of CD8α vs CD8β in CD8+ Tn, Tcm, Tem or Temra at baseline levels in order to understand the relative expression of the CD8α and CD8β chains after culture with IL-15. To that, we labeled fresh collected PBMC with antibodies against CCR7, CD45RA, CD8α and CD8β. As shown in **Figure 1F**, while CD8+ Tn cells are mainly constituted by CD8αβ^high^ T cells, CD8+ Tcm cells already showed an inversion of the proportions of CD8αβ^high^ vs. CD8αβ^low^ T cells. In contrast, in the CD8+ Tem and Temra pools, the percentage of CD8αβ^low^ T cells was predominant.

**Figure 2:**
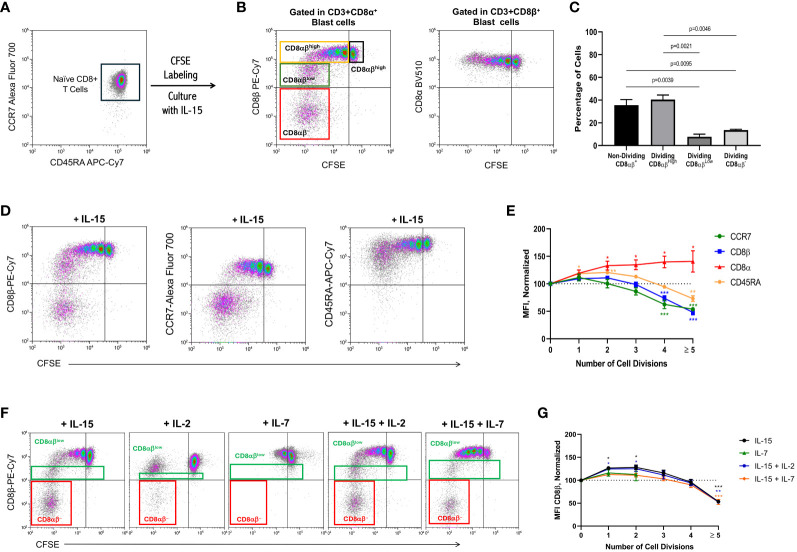
IL-15 induces down-modulation of CD8β in naïve CD8+ T cells. PBMC were isolated as described in the legend of **Figure 1**. Naïve CD8+ T cells were purified by using negative selection kits, labeled with CFSE and cultured with IL-15, IL-2, IL-7 or combinations of them (10 ng/mL) for 12 days. At the end of the culture, cells were harvested, washed, and approximately 0.5×10^6^ cells stained using fluorochrome-conjugated antibodies against CD3, CD8α, CD8β, CD45RA, CCR7 and CD56, and acquired in an Accuri C6 or a CytoFLEX flow cytometers. **(A)** Representative flow graph showing CD45RA vs. CCR7 expression on the negatively selected naïve CD8+ T cells (purity was usually higher than 97%) before CFSE-labeling and culture with IL-15. (**(B)** left dot-plot) Flow graph shows CFSE vs. CD8β expression after the 12-day culture period after gating on CD3^+^CD8α^+^ T cell blasts in order not to interfere with the analysis of the CD8β chain. Four CD8+ T cell populations can be distinguished: non-dividing CD8αβ^high^ (black rectangle), dividing CD8αβ^high^ (orange rectangle), dividing CD8αβ^low^ (green rectangle), and dividing CD8αβ^−^ (red rectangle). (**(B)**, right dot-plot). Flow graph shows CFSE vs. CD8α expression after the 12-day culture period after gating on CD3^+^CD8β^+^ T cell blasts in order not to interfere with the analysis of the CD8α chain. **(C)** Graph shows the percentage of the four different subsets framed in **Figure 2B** (left dot-plot, mean ± SEM, n=7). P values are indicated. **(D)** Dot-plots show CFSE halving vs. CD8β (left dot-plot), CCR7 (middle dot-plot) and CD45RA (right dot-plot) in CD8+ Tn cells after 12-day culture with IL-15. **(E)** Graph shows the normalized mean fluorescence intensity (MFI) values for the CD8α, CD8β, CCR7 and CD45RA receptors, calculated as indicated in the Material & Methods, from purified naïve CD8+ T cells that undergone none, one to 4, or >5 divisions, after culture with IL-15 for 12 days. Data are presented as the mean±SEM; n=6 for zero to 4 divisions, and n=3 for ≥ 5 divisions (for CD8α CD8β and CCR7) and n=2 for CD45RA. *p<0.05; ***p<0.001, as determined by one-way analysis of variance (ANOVA) with Dunnett’s *post hoc* test, comparing each cell division with cells that did not divide (0 divisions). **(F)** Dot-plots show CFSE halving vs. CD8β expression in CD8+ Tn cells after 12-day cultures with the indicated cytokines. Dividing CD8αβ^low^ (green rectangle) and dividing CD8αβ^−^ (**red** rectangle) are indicated. **(G)** Graph shows the normalized mean fluorescence intensity (MFI) values for CD8β, calculated as indicated in the Material & Methods, from purified naïve CD8+ T cells that undergone none, one to 4, or >5 divisions, after culture with IL-15, IL-7, IL15+IL-2 and IL-15+IL-7 for 12 days. Data are presented as the mean±SEM; n=2, *p<0.05; **p<0.01, ***p<0.001, as determined by one-way analysis of variance (ANOVA) with Dunnett’s *post hoc* test, comparing each cell division with cells that did not divide (0 divisions).

To avoid the possible confounding effect of the presence of NK cells on the PBMC preparations and to ascertain whether the CD8β chain downmodulation could also be seen in CD8+ Tn cells, these were purified from PBMC preparations using negative isolation kits. The isolated CD8+ Tn cells were CD45RA+CCR7+ and purity was usually higher than 97% (**Figure 2A**). These experiments showed that IL-15 also induced a cell-cycle dependent down-modulation of the CD8β chain in CD8+ Tn cells, leading to the generation of a pool of CD8αβ^low^ T cells and a pool of CD8β^−^ T cells, namely in cells that divided ≥5 times (**Figure 2B**, left dot-plot). In marked contrast, expression of the CD8α chain remained constant or increased slightly with each cycle of cell division (**Figure 2B**, right dot-plot). Similar results were obtained with total bulk CD8+ T cells (see **Supplementary Figure 2**). The percentage of the four CD8+ T cell subsets framed on **Figure 2B** are illustrated in **Figure 2C**. Simultaneous analysis of CCR7 and CD45RA expression demonstrated that the CD8β down-modulation seen in the most dividing IL-15-activated CD8+ T cells was paralleled by a similar down-modulation of the naïve chemokine receptor CCR7 and the generation of CD8+CCR7− T cells. In contrast, although the CD45RA receptor was also downmodulated by IL-15, no CD8+CD45RA− were observed (**Figure 2D**), which is in accord with the fact that IL-15 stimulation drives CD8+ Tn cells toward the acquisition of a highly differentiated phenotype (19, 20). **Figure 2E** summarizes the results of the expression of CD8α, CD8β, CCR7 and CD45RA in CD8+ Tn samples after culture with IL-15. The normalized MFI values (see Material & Methods) for the expression of CD8β and CCR7 paralleled each other and started to decrease significantly after the 4^th^ cycle of cell division (p<0.001). Regarding the normalized MFI values for CD45RA expression, an initial increase in the 1^st^ to 3^rd^ division cycles was followed by a slight downmodulation after 5^th^ cycle of cell division (p<0.01). In contrast, the normalized MFI values for the expression of CD8α increased, being statistically significantly higher at cell division cycles 2, 3, 4 and ≥5 (p<0.05).

Next, we wanted to ascertain whether the effect of IL-15 can also be seen with other γc cytokines, such as IL-2 and IL-7, which have been used for expanding CD8+ T cells for experimental and clinical therapeutic purposes. As shown in **Figure 2F**, IL-2 neither induced CD8αβ^low^ T cells nor a significant pool of CD8αα T cells when compared to IL-15 (3.8 ± 0.5 vs. 14.6 ± 0.7, mean ± SEM, p=0.0027). Indeed, the number of CD8+ T cells entering successive cell divisions was minimal when compared to IL-15 or the combination of IL-15+IL-2 and IL-15+IL-7 (see **Supplementary Table 3**). Of note, IL-7 was only capable to drive naïve CD8+ T cells into two cycles of cell division and, consequently, no CD8β down-modulation was observed (see **Supplementary Table 3**). Finally, the combined use of these cytokines showed that IL-15+IL-2 did not significantly affect the percentage of CD8αα T cells when compared to IL-15 alone (16.1 ± 2.2 vs. 14.6 ± 0.7, p=0.7578). Although the use of IL-15+IL-7 slightly inhibited the formation of CD8αα T cells when compared to IL-15 alone, the difference was not statistically significant (9.7 ± 0.4 vs. 14.6 ± 0.7, p=0.0704). **Figure 2G** summarizes the results of the expression of CD8β in CD8+ Tn samples after culture with IL-15.

### IL-15 preferentially down-modulates the CD8β M-4 isoform

**Figure 3:**
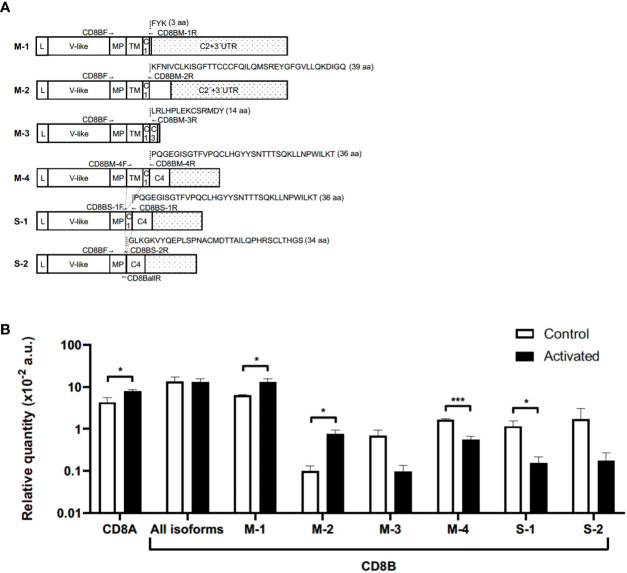
Expression of *CD8A* and *CD8B* genes in naïve CD8+ T cells before and after culture with IL-15. **(A)** Schematic representation of *CD8B* studied isoforms, the location of primers used for qPCR, and respective unique C-terminal amino acid sequences. White squares and doted squares represent exons and 3’UTR, respectively. For more information about *CD8B* isoforms see **Supplementary Table 2**; **Figure 3**. **(B)** Relative mRNA expression levels normalized to the geometric mean of two reference genes (*GAPDH* and *RPS18S*) multiplied by 100. Graphed qPCR data represents the mean relative quantity of four experiments ± SEM in naïve CD8+ T cells before (Control) and after (Activated) culture with IL-15 for 12 days. *p<0.05; ***p<0.001, Mann-Whitney U test. aa, amino acids; a.u., arbitrary units; C, Cytoplasmic; IgV-like, Immunoglobulin V-like; L, Leader peptide; MP, membrane proximal; TM, transmembrane; UTR, untranslated region;.

Next, we wanted to ascertain whether the results of CD8β down-modulation were related, or not, with the expression levels of any of the CD8β mRNA isoforms described. For that, we performed qPCR using specific primers designed to amplify the six described CD8β mRNAs isoforms (**Figure 3**; **Supplementary Table 2**): four membrane-anchored (M-1, M-2, M-3 and M-4) and two secreted (S-1 and S-2) in CD8+ Tn cells before and after activation with IL-15 (see **Figure 3A** for exons’ nomenclature and for visualization of primers location). As summarized in **Figure 3B**, the mRNA levels of the isoforms M-1 and M-2 were significantly increased in IL-15-activated CD8+ T cells (p<0.05). In marked contrast, the mRNA level for the M-4 isoform was significantly decreased (p<0.001). Although the mRNA for the M-3 isoform was also decreased, it did not reach statistical significance. Interestingly, we detected the two soluble isoforms described earlier (S-1 and S-2), with the mRNA of the former being significantly decreased (p<0.05). Also illustrated in **Figure 3** are the mRNA levels for all CD8β isoforms, which remained constant, and the mRNA levels for the CD8α chain, which was significantly increased in IL-15 activated CD8+ Tn cells (p<0.05), in accordance with the results of cell surface CD8α expression (see **Supplementary Figure 3** for a representative gel).

### IL-15 increases the amount of Lck in IL-15-activated CD8+ T cells

**Figure 4:**
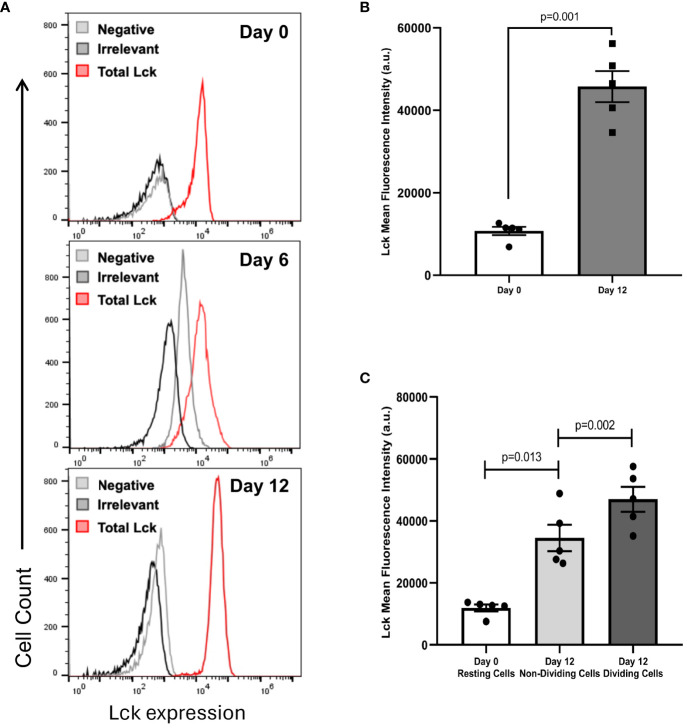
IL-15 increases the amount of total Lck in CD8+ T cells. PBMC were isolated as described in the legend of **Figure 1**. Total CD8+ T cells were purified using negative selection kits, labeled with CFSE and cultured with IL-15 (10 ng/mL) for 12 days. Approximately 0.5×10^6^ CD8+ T cells at day 0 and at day 12 were stained using fluorochrome-conjugated antibodies against CD3 and CD8α, or CD3 and CD8β. Then, labeled CD8+ T cells were fixed, permeabilized and incubated with fluorochrome-conjugated antibodies against total Lck and irrelevant IgG, as indicated in Materials and Methods, and acquired in a BD Accuri C6 flow cytometer. **(A)** Representative histograms showing the fluorescence emission in freshly isolated CD8+ T cells after incubation with anti-Lck, irrelevant IgG, or none at day 0, 6 and 12. **(B)** Graph showing the amount of total Lck, as determined by the MFI values obtained after subtracting background fluorescence obtained with the irrelevant IgG, at day 0, 6 and 12. Data are presented as the mean±SEM; n=5. Paired samples T-test was used to assess the significance of the differences in MFI values between days 0 and 12. **(C)** Graph showing the amount of total Lck, as determined by the MFI values obtained after subtracting background fluorescence obtained with the irrelevant IgG, at days 0 (Resting Cells), 12 (Non-Dividing Cells) and 12 (Dividing Cells). Data are presented as the mean±SEM; n=5. Paired samples T-test was used to assess the significance of the differences in MFI values between days 0 and 12.

Considering the importance of the tyrosine kinase Lck in the transmission of intracellular activation signals in human CD8+ T cells, we wanted to ascertain if the levels of total Lck changed between freshly isolated CD8+ T cells and differentiated CD8+ T cells after 12 days in culture with IL-15. By using total CD8+ T cells, an increase in the amount of Lck, as determined by the mean fluorescence intensity (MFI), was consistently observed between resting CD8+ T cells at day 0 and CD8+ T cell blasts at day 12 in all the experiments performed (compare **Figures 4A, B**). As shown in **Figure 4C**, on average, the MFI values of Lck observed between CD8+ T cells at day 0 and CD8+ T cell blasts at day 12 increased by 4.5-fold (10728 ± 996 vs. 45739 ± 3778, mean ± SEM, p=0.001). Kinetic studies at day 0, 6 and 12, showed a time-dependent increase in Lck expression, as determined by MFI values, from day 0, to day 6, to day 12 (13078 vs.16846 vs. 47133, respectively). Next, we performed an analysis of Lck expression in CD8+ T cells according to their dividing status, that is CD8+ T cells at day 0 (resting cells, RC), CD8+ T cells that survived the 12-day culture with IL-15 but did not divide (non-dividing cells, NDC), and CD8+ T cells that entered successive cycles of cell division (dividing cells, DC). As shown in **Figure 4D**, the analysis showed that there was a gradual increase in the amount of Lck, as determined by the MFI values, from RC to NDC (10708 vs 33299, p=0.013, n=5) and from NDC to DC (33299 vs. 45739, p=0.002, n=5).

## Discussion

Apart from initial reports describing the expression of CD8β in human T cells (13, 14, 26–30), studies examining factors and molecular mechanisms regulating the expression of the CD8αβ receptor in humans are scarce. Thus, with the exception of one study using retrovirally transduced peripheral blood cells and cell lines that showed a Nef-mediated down-modulation of the CD8β chain (31), most other studies in humans have been performed with *ex vivo* blood lymphocytes from healthy blood donors (4, 5, 32), diseased patients (33–35), and virus-infected patients (6, 7). More recently, we have shown that the frequency of CD3+CD8β^low^ T cells in peripheral blood of elderly people with cognitive impairment is decreased when compared to age-matched people without cognitive impairment, suggesting a possible protective role for CD3+CD8β^low^ T cells against cognitive decline and neurodegeneration (34). Importantly, some of these studies showed that down-modulation or absence of the CD8β chain was associated with the generation of CD8+ T cells with an effector-memory phenotype, and defined by lack of CD28, and expression of CD8αα homodimers, perforin, granzymes, and IFN-γ (4–6, 32).

Our work has extended these studies allowing us to conclude that *in vitro*, IL-15 is a novel factor that contributes to the formation of a pool of conventional CD8αα T cells. Intriguingly, the decrease in the expression of total CD8β protein on the surface and that results in the generation of a pool of CD8αα T cells was not paralleled by a decrease in total mRNA (all isoforms). Therefore, in principle, the cell surface CD8β decrease cannot be explained solely by the IL-15-induced decrease of the M-4 isoform, unless there is some sort of mechanism where one of the mRNA forms is less stable or is less efficiently translated into protein. In that regard, the M-2 protein isoform has been shown to be ubiquitinylated, which directs the CD8β protein to the lysosomal compartment where it can be degraded (15). Thus, it is a likely possibility that the marked increase in mRNA for the M-2 isoform does not result in a net increase in CD8β protein at the plasma membrane. Nevertheless, these results show for the first time that IL-15 is a key cytokine that, *in vitro*, prompts the generation of conventional human CD8αβ^low^ and CD8αα T cells previously reported, like CD8+ Temra cells, to exist *in vivo* (5, 6, 21). These results also reinforce the notion that IL-15 is a key cytokine that is capable of pushing naïve CD8+ T cells toward a more differentiated functional phenotype, characterized by loss of naïve cell surface receptors and *de novo* expression of NK-like receptors, as demonstrated by several groups, including ours (19, 36, 37). Interestingly, in the latter work, a significant decrease in the expression of the CD8β gene in CD8+ TEMRA cells when compared to CD8+ TN (-2,45-fold, p=0.000024, ANOVA) was found, which may be an indication that a pool of CD8+ TEMRA cells found in peripheral blood may actually contain CD8αα+αβ^low^ and/or CD8αα+αβ^–^ (37).

Importantly, our results have shown that even the CFSE halving between CD8αβ^low^ and CD8αα is comparable, their CD8β expression are different, i.e., the latter population has completely lost CD8β expression. In our view, *in vitro* IL-15-activated naïve CD8+ T cells enter successive cycles of cell division and, at some point around the 5^th^ to 6^th^ cycle the dividing CD8+ T cells start to diminish CD8β chain, with a sizeable fraction of these CD8+ T cells losing completely expression. One possibility explaining this novel result is that a subset of the most dividing CD8+ T cells is more susceptible to the epigenetic modifications induced by IL-15 signaling and result in the silencing the gene(s) coding for the CD8β chain(s). This view is not unlikely since a recent study has shown that IL- 15, via STAT3 and STAT5 signaling, mediated silencing of Epstein-Barr Nuclear Antigens (EBNA) via epigenetic effects (38).

The novel findings reported in this study are important because recent studies have identified innate-like CD8αα T cells endowed with Treg functions and capable of controlling effector T cell responses in human peripheral blood (39), suggesting that expression of CD8αα homodimers, besides being a feature of expanded effector-memory CD8+ Temra cells, endows these CD8+ T cells with regulatory functions. Thus, in mice models, CD8αα receptors induce the expression of pro-survival molecules such as IL-7Rα and Bcl-2 (40). CD8αα receptors have also been proposed as repressors that negatively regulate CD8+ T cell activation (41), which is compatible with their pro-survival function. Moreover, a predominant expression of CD8αα homodimers by IL-15-induced CD8αβ^−^ T cells may favor preferential *cis*-interactions with other receptors expressed by effector-memory CD8+ T cells, including KIR3DL1 (42), MHC class I molecules (43) and β2m-free heavy MHC class I chains, also known as open MHC-I conformers (44–46). In either case, the *cis*-interactions would regulate the CD8+ T cell responses.

Importantly, we have also shown that IL-15-activated CD8+ T cells markedly augmented the amount of the total amount of the tyrosine kinase Lck. In view of recent data by the Gascoigne group showing that free Lck is more active than the fraction of CD8α-bound Lck (47), these results may have implications at the signaling and responsive levels of the differentiated CD8αα T cells. Based on our results and the existing experimental data, it can be proposed a scenario where the CD8αα^+^αβ^low^ and CD8αα^+^αβ^−^ T cells would be, like CD8+ Temra cells, less responsive to TCR-mediated signals, an issue that warrants further investigations. In any case, this work highlights the fact that the capacity of IL-15 to induce CD8+CD28- Temra cells *in vitro* and *in vivo* cannot be dissociated from the loss of the CD8β chain. In summary, these are novel findings that may have physiological relevance in settings where the plasma and tissue levels of IL-15 are increased. These settings include hematologic and solid tumors, autoimmune diseases, HIV infection, neurodegenerative disorders, and also exercise training in healthy subjects (48–53). In the latter situation, an inverse relationship between IL-15 and adipose tissue mass indexes was observed, suggesting that IL-15 has beneficial metabolic activities in obesity and type 2 diabetes. Whether these beneficial effects are mediated by CD8+ Temra and/or CD8αα T cells are issues that warrant further investigations.

## Data availability statement

The original contributions presented in the study are included in the article/**Supplementary Material**. Further inquiries can be directed to the corresponding author.

## Ethics statement

The studies involving humans were approved by Ethics Committee of the University of Beira Interior (Ref. Number CE-UBI-Pj-2017-012). The studies were conducted in accordance with the local legislation and institutional requirements. The participants provided their written informed consent to participate in this study.

## Author contributions

FA conceived the study, analyzed data, and wrote the manuscript; AE performed experiments, analyzed data and wrote the manuscript; DR-F performed experiments, analyzed data and reviewed the manuscript; EC analyzed data, performed statistical analysis, and reviewed the manuscript. MU and SW supervised experiments performed in Düsseldorf and reviewed the manuscript. All authors contributed to the article and approved the submitted version.

## Supplementary material

Supplementary Figure 1 Quantification of cell divisions. Representative dot-plot of CFSE fluorescence halving (X axis) vs. CD8β expression (Y axis) relative to one representative experiment and illustrating how the regions were created electronically to determine the mean fluorescence intensity (MFI) values in each cycle of cell division, as indicated in the Material and Methods.

Supplementary Figure 2 Effect of IL-15 on CD8β expression in bulk CD8+ T cells. Total CD8+ T cells were isolated by using negative isolation kits and cell cultured for 12 days in the presence of IL-15 as indicated in the legend of **Figure 2**. At the end of the culture, cells were harvested, washed, and approximately 0.5×10^6^ cells were stained with fluorochrome-conjugated antibodies against CD3, CD8α, and CD8β and acquired in an Accuri C6 flow cytometer. An electronic region was created around CD3+CD8α+ blast cells, which were subsequently analyzed for CFSE fluorescence halving and vs. CD8β expression. Results from two experiments **(A, B)** are shown.

Supplementary Figure 3 PCR products of selected gene transcripts. Photograph from a 2% (w/v) agarose gel from a representative sample of non-activated (CTR) and activated (ACT) naïve CD8+ T cells, showing the PCR bands of selected membrane CD8β isoforms (M-1, M-2, M-4), all CD8β, CD8α and the two reference genes (GAPDH and RPS18) used. On the left it is shown the molecular wight markers (MWM).
