# Supplementary figures and images for: In vitro IL-15-activated human naïve CD8+ T cells down-modulate the CD8β chain and become CD8αα T cells
Source: Front Immunol. 2024 Jun 5;15:1252439. doi: 10.3389/fimmu.2024.1252439 (PMC11188365; doi:10.3389/fimmu.2024.1252439)

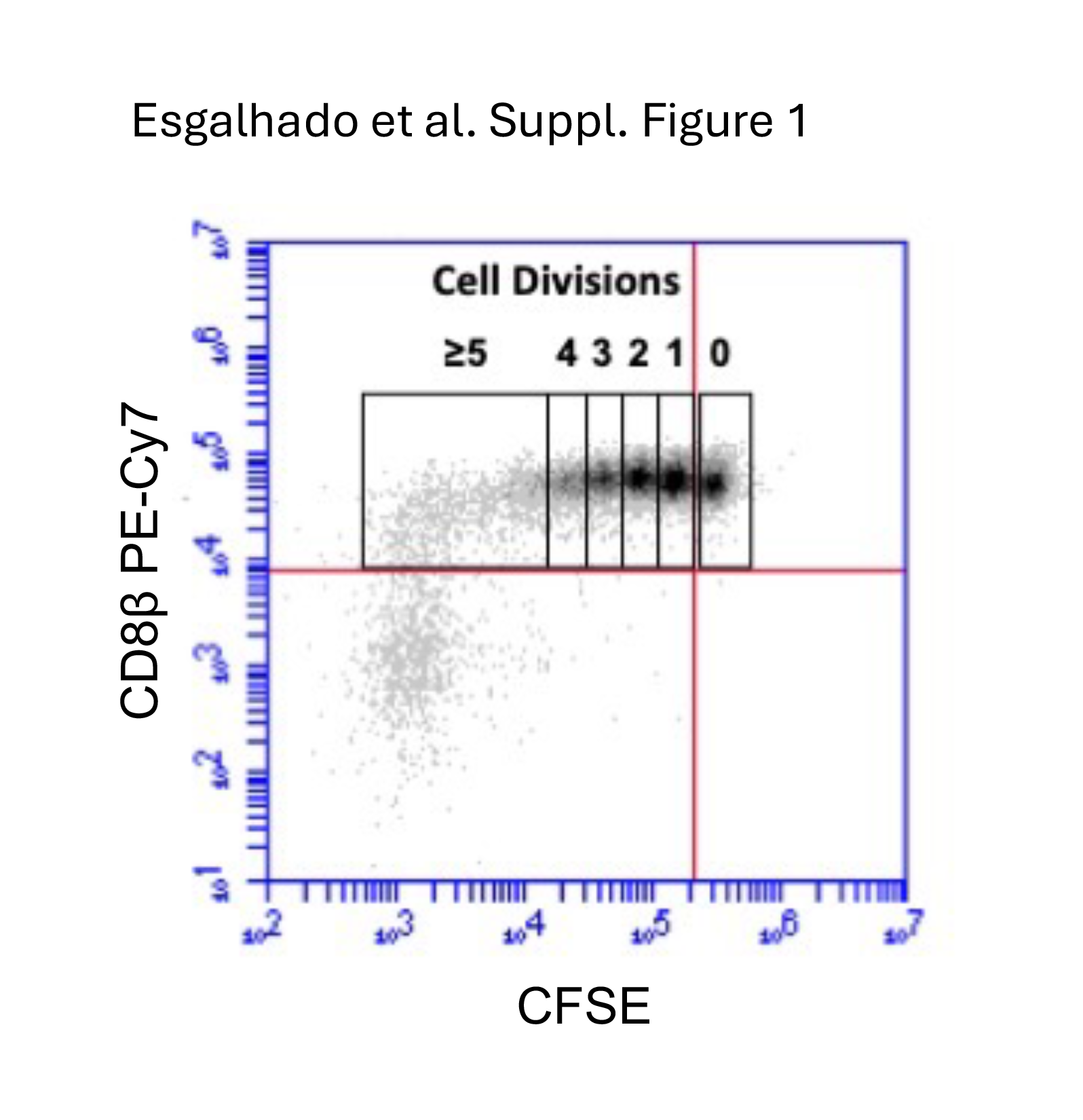

Supplement: Supplementary Figure 1 — Quantification of cell divisions. Representative dot-plot of CFSE fluorescence halving (X axis) vs. CD8β expression (Y axis) relative to one representative experiment and illustrating how the regions were created electronically to determine the mean fluorescence intensity (MFI) values in each cycle of cell division, as indicated in the Material and Methods. [file Image_1.tif]

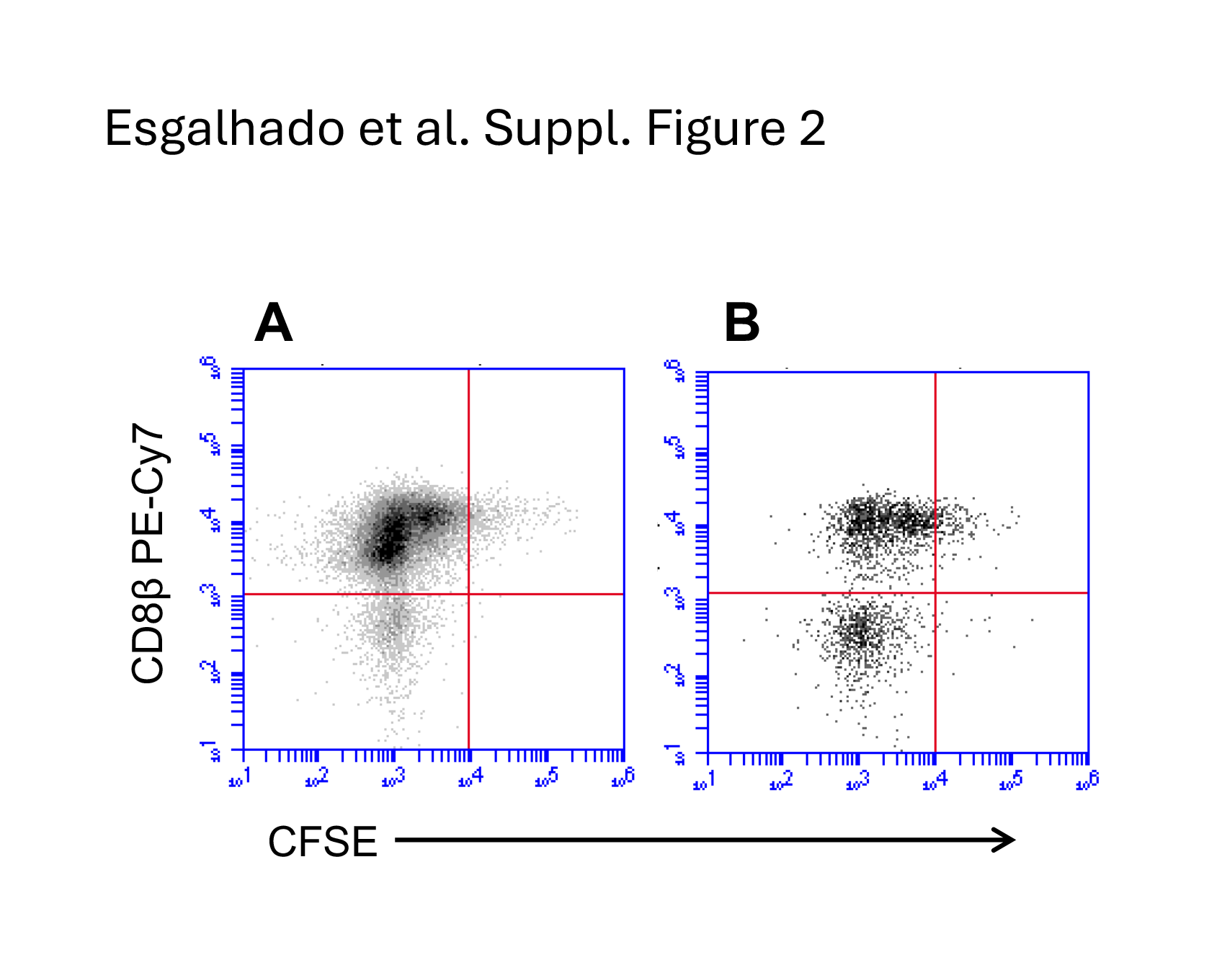

Supplement: Supplementary Figure 2 — Effect of IL-15 on CD8β expression in bulk CD8+ T cells. Total CD8+ T cells were isolated by using negative isolation kits and cell cultured for 12 days in the presence of IL-15 as indicated in the legend of Figure 2 . At the end of the culture, cells were harvested, washed, and approximately 0.5×10^6^ cells were stained with fluorochrome-conjugated antibodies against CD3, CD8α, and CD8β and acquired in an Accuri C6 flow cytometer. An electronic region was created around CD3+CD8α+ blast cells, which were subsequently analyzed for CFSE fluorescence halving and vs. CD8β expression. Results from two experiments (A, B) are shown. [file Image_2.tif]

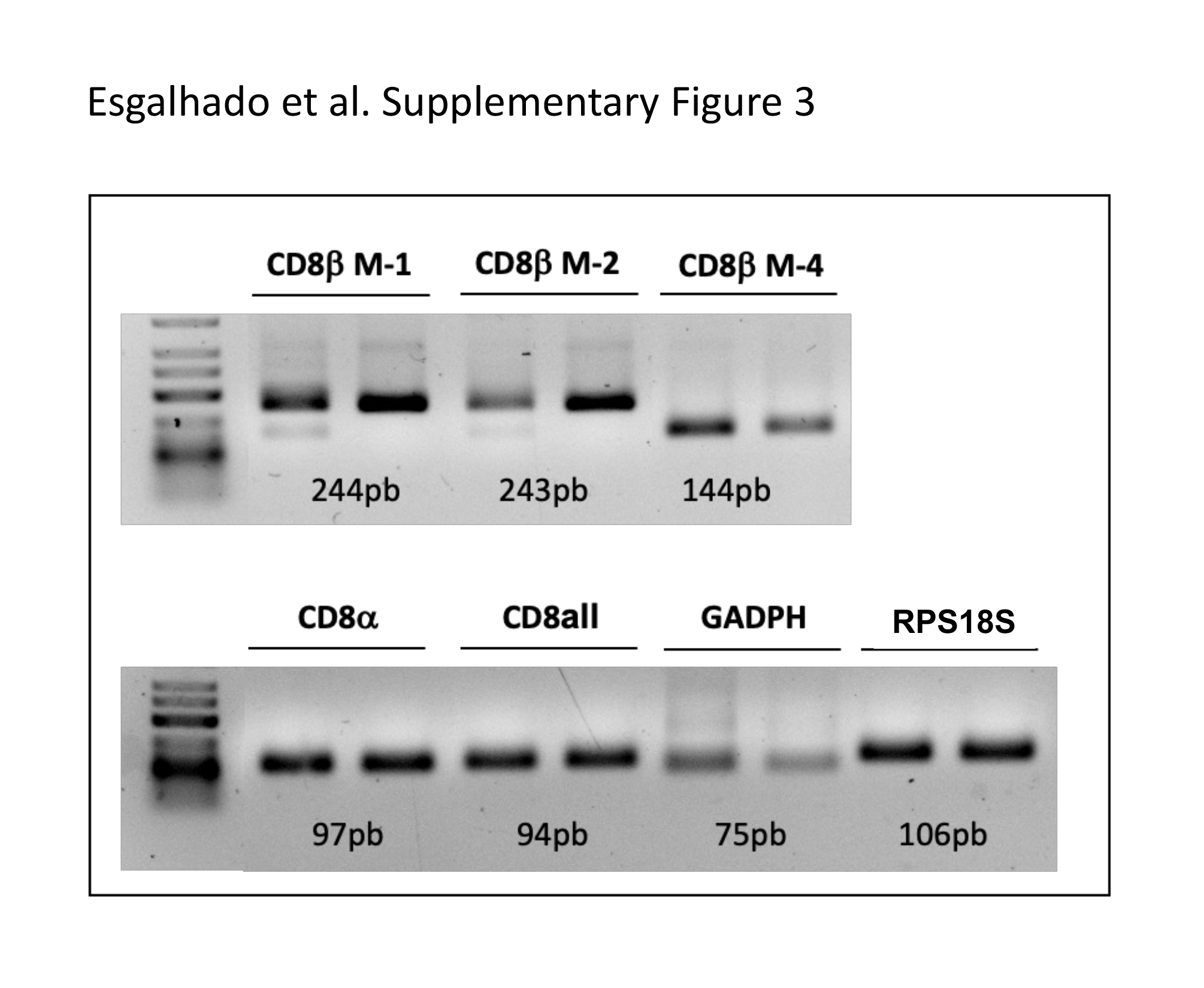

Supplement: Supplementary Figure 3 — PCR products of selected gene transcripts. Photograph from a 2% (w/v) agarose gel from a representative sample of non-activated (CTR) and activated (ACT) naïve CD8+ T cells, showing the PCR bands of selected membrane CD8β isoforms (M-1, M-2, M-4), all CD8β, CD8α and the two reference genes (GAPDH and RPS18) used. On the left it is shown the molecular wight markers (MWM). [file Image_3.tif]
