# Supplementary material for: In vitro IL-15-activated human naïve CD8+ T cells down-modulate the CD8β chain and become CD8αα T cells
Source: Front Immunol. 2024 Jun 5;15:1252439. doi: 10.3389/fimmu.2024.1252439 (PMC11188365; doi:10.3389/fimmu.2024.1252439)
Supplement: Supplementary file 4 [file Table_1.pdf]

Esgalhado et al. Supplemental Table 1. List of antibodies used.

Antibody	Conjugate	Clone	Isotype	Company
CD3	PE-Cy5	UCHT1	IgG1	BioLegend
CD3	APC	OKT3	IgG2a	BioLegend
CD4	PerCP-Cy5.5	RPA-T4	IgG1	BioLegend
CD8α	FITC	SK1	IgG1	BioLegend
CD8α	PE	RPA-T8	IgG1	BioLegend
CD8α	PE	OKT8	IgG2a	Thermo Fisher Scientific
CD8α	PerCP-Cy5.5	SK1	IgG1	BioLegend
CD8α	APC	SK1	IgG1	BioLegend
CD8α	Brilliant Violet 510™	RPA-T8	IgG1	BioLegend
CD8β	PE-Cy7	SIDI8BEE	IgG1	Thermo Fisher Scientific
CD28	PE	CD28.2	IgG1	BioLegend
CD45RA	FITC	HI100	IgG2b	BioLegend
CD45RA	APC-Cy7	HI100	IgG2b	BioLegend
CD56	PE	HCD56	IgG1	BioLegend
CD56	Brillian Violet 650™	HCD56	IgG1	BioLegend
CD197 (CCR7)	PE	G043H7	IgG2a	BioLegend
CD197 (CCR7)	Alexa Fluor® 700	150503	IgG2a	BD Biosciences
Mouse IgG	Alexa Fluor® 647	MOPC-21	IgG1	BioLegend
Lck	Alexa Fluor® 647	LCK-01	IgG1	BioLegend
