# Supplementary material for: In vitro IL-15-activated human naïve CD8+ T cells down-modulate the CD8β chain and become CD8αα T cells
Source: Front Immunol. 2024 Jun 5;15:1252439. doi: 10.3389/fimmu.2024.1252439 (PMC11188365; doi:10.3389/fimmu.2024.1252439)
Supplement: Supplementary file 5 [file Table_2.pdf]

Esgalhado et al, Supplemental Table 2. Set of primers used in qPCR.

Gene	Name in this paper	Name and/or transcript variants	SeqRef Accession number	Primer sequence (5'→3')	Amplicon length (bp)	Location#
CD8A	CD8A	CD8α molecule; all mRNA transcript variants*	See footnote	F: TGAGCAACTCCATCATGTACTTCAG<br>R: GGCGCCGGTGTTGGT	97	Exon IgV-like<br>Exon MP
CD8B	All isoforms	CD8β molecule; transcript variants X1, 2, 3, 4, 5 & 6**	See variants below	F: GTGTGGTTGATTTCCTTCCCA<br>R: TTCTGGGTCTCTGGCCTGG	94	Spanning exons IgV-like & MP<br>MP exon
	M-1	CD8β molecule/transcript variant 5	NM_004931.5	F: GTGTGGTTGATTTCCTTCCCA<br>R: TTCTCTGCTCATTTGTAAAATTGTTTC	244	Spanning exons IgV-like & MP<br>Spanning exons C1 and C2
	M-2	CD8β molecule/ transcript variant X1	XM_011533164.3	F: GTGTGGTTGATTTCCTTCCCA<br>R: GGCAAACGATATTGAATTTCTGTTTC	243	Spanning exons IgV-like & MP<br>Spanning exons C1 and C2'
	M-3	CD8β molecule/ transcript variant 3	NM_172101.5	F: GTGTGGTTGATTTCCTTCCCA<br>R: TTCTGGAACATTTCTCCAGTGG	258	Spanning exons IgV-like & MP<br>C3 exon
	M-4	CD8β molecule/ transcript variant 2	NM_172213.5	F: CCCACTTTGTAGCCCCATCA<br>R: ATACCTTCCCCTTGAGGCTGTT	144	TM exon<br>Spanning exons C1 and C4
	S-1	CD8β molecule/ transcript variant 4	NM_172102.5	F: CAGAGACCCAGAAGGGCCGG<br>R: ATACCTTCCCCTTGAGGCTGTT	70	Spanning exons MP & C1<br>Spanning exons C1 & C4
	S-2	CD8β molecule/ transcript variant 6	NM_001178100.2	F: GTGTGGTTGATTTCCTTCCCA<br>R: ATACCTTCCCCTTGAGGCCCTT	114	Spanning exons IgV-like & MP<br>Spanning exons MP & C4
GAPDH	GAPDH	glyceraldehyde-3-phosphate dehydrogenase***	See footnote	F: CGCCAGCCGAGCCACATC<br>R: CGCCCAATACGACCAAATCCG	76	Spanning exons 1 & 2<br>Spanning exons 2 & 3
RPS18	RPS18	Homo sapiens ribosomal protein S18 (RPS18)	NM_022551.3	F: CAGAATCCACGCCAGTACAAG<br>R: GCTTGTTGTCCAGACCATTG	106	Exon 4<br>Exon 5

F, forward; R, reverse

*Transcript variants 1, 2, 3 & 5 (NM_001768.7; NM_171827.4; NM_001145873.1; NM_001382698.1)

**Also, would amplify *CD8B2*. However, this *CD8B* paralog has been shown to lose expression specifically in T cells and to gain expression in brain tissues (cortex) (Doughert et al, 2018).

***Transcript variants 1, 3, 4 & 7 (NM_002046.7; NM_001289745.3; NM_001289746.2; NM_001357943.2)

See Figure 3A for exons' nomenclature and for visualization of primers location. C2' results from an alternative splicing acceptor site of exon C2, originating the predicted CD8β molecule (CD8B), transcript variant X.
