# Supplementary material for: In vitro IL-15-activated human naïve CD8+ T cells down-modulate the CD8β chain and become CD8αα T cells
Source: Front Immunol. 2024 Jun 5;15:1252439. doi: 10.3389/fimmu.2024.1252439 (PMC11188365; doi:10.3389/fimmu.2024.1252439)
Supplement: Supplementary file 6 [file Table_3.pdf]

Esgalhado et al, Supplemental Table 3.

Events (CD8αβ+ T cells) gated in every cell cycle of cell division after culture with the different cytokines

	**IL-15**						
	**0 Div.**	**1 Div.**	**2 Div.**	**3 Div.**	**4 Div.**	**≥ 5 Div.**	**TOTAL**
**Exp#1**	6494	3957	1934	1521	889	2518	17313
**Exp#2**	9407	4618	2003	895	493	1905	19321
**Mean**	**7951**	**4288**	**1969**	**1208**	**691**	**2212**	**18317**
	**IL-2**						
	**0 Div.**	**1 Div.**	**2 Div.**	**3 Div.**	**4 Div.**	**≥ 5 Div.**	**TOTAL**
**Exp#1**	16120	196	25	37	82	3067	19527
**Exp#2**	18394	160	24	44	74	291	18987
**Mean**	**17257**	**178**	**25**	**41**	**78**	**1679**	**19257**
	**IL-7**						
	**0 Div.**	**1 Div.**	**2 Div.**	**3 Div.**	**4 Div.**	**≥ 5 Div.**	**TOTAL**
**Exp#1**	10686	7311	1432	ND	ND	ND	19429
**Exp#2**	18721	1506	70	ND	ND	ND	20297
**Mean**	**14704**	**4409**	**751**	ND	ND	ND	**19863**
	**IL-15 + IL-2**						
	**0 Div.**	**1 Div.**	**2 Div.**	**3 Div.**	**4 Div.**	**≥ 5 Div.**	**TOTAL**
**Exp#1**	7077	4321	2049	1458	906	2525	18336
**Exp#2**	7840	3933	1622	776	445	2408	17024
**Mean**	**7459**	**4127**	**1836**	**1117**	**676**	**2467**	**17680**
	**IL-15 + IL-7**						
	**0 Div.**	**1 Div.**	**2 Div.**	**3 Div.**	**4 Div.**	**≥ 5 Div.**	**TOTAL**
**Exp#1**	2007	3942	4603	4324	2947	1497	19320
**Exp#2**	2770	4861	4996	4166	1329	1397	19519
**Mean**	**2389**	**4402**	**4800**	**4245**	**2138**	**1447**	**19420**
**ND, Not Detectable**							
